# Correlation between Saliva Levels and Serum Levels of Free Uremic Toxins in Healthy Volunteers

**DOI:** 10.3390/toxins15020150

**Published:** 2023-02-13

**Authors:** Nicolas Fabresse, Islam Amine Larabi, Emuri Abe, Elodie Lamy, Claire Rigothier, Ziad A. Massy, Jean-Claude Alvarez

**Affiliations:** 1 Laboratory of Pharmacokinetics and Toxicology, La Timone University Hospital, 264 Rue Saint Pierre, CEDEX 5, 13385 Marseille, France; 2 Department of Pharmacology and Toxicology, Raymond Poincaré Hospital, AP-HP, 104 Boulevard Raymond Poincaré, 92380 Garches, France; 3 U-1018, CESP-Inserm, MOODS Team, Simone Veil Medical School, Versailles Saint-Quentin-En-Yvelines (UVSQ)-Paris-Saclay University, 2 Av. de la Source de la Bièvre, 78180 Montigny-le-Bretonneux, France; 4 MasSpecLab, Inserm U-1173, Simone Veil Medical School, Versailles Saint-Quentin-En-Yvelines (UVSQ)-Paris-Saclay University, 2 Av. de la Source de la Bièvre, 78180 Montigny-le-Bretonneux, France; 5 Service de Néphrologie, Transplantation, Dialyse et Aphérèses, CHU de Bordeaux, 33000 Bordeaux, France; 6 Service de Néphrologie, CHU Ambroise Paré, Assistance Publique—Hôpitaux de Paris & Université Paris-Saclay (Versailles-Saint-Quentin-en-Yvelines), 9 Avenue Charles de Gaulle, 92104 Boulogne Billancourt, France; 7 Inserm U-1018 Centre de Recherche en Épidémiologie et Santé des Populations (CESP), Équipe 5, 94807 Villejuif, France

**Keywords:** uremic toxins, saliva, indoxyl sulfate, p-cresyl sulfate, mass spectrometry

## Abstract

The objective of the present study was to investigate the putative correlation between the saliva concentration and free serum concentration for 10 uremic toxins (UTs; eight protein-bound solutes: 3-carboxy-4-methyl-5-propyl-2-furanpropanoic acid (CMPF), hippuric acid (HA), indole-3-acetic acid (3-IAA), indoxyl sulfate (I3S), kynurenic acid (KA), kynurenine (KYN), p-cresyl glucuronide (pCG), and p-cresyl sulfate (pCS); two free, water-soluble, low-molecular weight solutes: phenylacetylglutamine (PAGN) and trimethylamine N-oxide (TMAO); and three precursors: tyrosine (Tyr), phenylalanine, and tryptophan). Saliva samples and blood samples were collected simultaneously from 18 healthy volunteers. After the addition of internal standards, 50 µL of saliva or serum were precipitated with methanol. UTs and precursors were quantified using a validated LC-MS/MS method. The saliva–serum correlation was statistically significant (according to Spearman’s coefficient) for six UTs (TMAO, HA, I3S, pCS, 3-IAA, and CMPF). Tyr presented a weak saliva-serum correlation (*p* = 0.08), whereas the other two precursors did not show a saliva–serum correlation. For three UTs (KYN, KA and pCG), we were unable to test the correlation since the saliva or serum levels were too low in many of the volunteers. The present study is the first to report on the saliva concentrations of TMAO, KYN, HA, PAGN, pCG, and 3-IAA.

## 1. Introduction

Chronic kidney disease (CKD) is defined as the presence of kidney damage or an estimated glomerular filtration rate (eGFR) below 60 mL/min/1.73 m^2^ for 3 months or more—irrespective of the cause [1]. Patients with CKD retain a variety of solutes, which have been classified into three groups: free small water-soluble solutes (molecular weight MW < 500 Da), middle molecules (MW > 500 Da), and protein-bound solutes [2]. These compounds are called “uremic retention solutes” or, when they interact negatively with biological functions, “uremic toxins” (UTs) [3]. It has recently been suggested that these UTs have a role in the genesis of CKD complications and/or comorbidities, such as endothelial dysfunction, immune dysfunction, aortic calcification, and cardiovascular mortality [4]. Hence, UT levels must be monitored, to protect patients from disease progression and the corresponding negative outcomes.

In CKD patients, UT concentrations are usually measured in the serum. However, these molecules are bound to plasma proteins to different extents, and so only the free fraction is able to diffuse into the tissues and exert adverse effects. Monitoring the free fraction of plasma UTs requires complicated, tedious, and expensive methods (e.g., ultrafiltration and equilibrium dialysis) that are not easy to implement for routine use. In recent years, however, saliva has aroused growing interest as an alternative diagnostic matrix because the free UT fraction can cross biological membranes. Saliva offers several advantages, relative to the blood: the sampling is quick, simple, non-invasive and non-stressful, does not generate lesions, generates a sufficient volume, and can be repeated if necessary. A correlation between the serum concentration and the saliva concentration has been observed for certain drugs (carbamazepine and phenytoin) and endogenous substances (cortisol and creatinine) [5,6,7,8]. Recently, Korytowska et al. found a correlation between the P-cresyl sulfate (PCS) and indoxyl sulfate in saliva and the biologically active fractions in serum [9].

The objective of the present study was to (i) validate the saliva-based adaptation of a liquid chromatography-tandem mass spectrometry (LC-MS/MS) assay originally developed for quantifying 10 UTs (eight protein-bound solutes: hippuric acid, 3-carboxy-4-methyl-5-propyl-2-furanpropanoic acid (CMPF), indole-3-acetic acid, indoxyl sulfate (IS), kynurenic acid, kynurenine, p-cresyl glucuronide, p-cresyl sulfate (PCS) and two free water-soluble, low-molecular weight solutes: phenylacetylglutamine and trimethylamine N-oxide (TMAO)) and three precursors (tyrosine, tryptophan, and phenylalanine) in serum, and (ii) to evaluate the correlation between each compound’s respective free saliva and serum levels.

## 2. Results and Discussion

Since UTs are endogenous compounds normally present in saliva, the method was validated by using a combination of aqueous calibration curves, quality controls, and isotope-labelled internal standards (i.e., deuterated analogs with very similar physicochemical properties) (Supplementary Document S1). The characteristics of the validated method have been published previously [10].

The data on linearity, the limit of detection, and the matrix effect for the 10 UTs and the three precursors in saliva are summarized in Table 1. None of the compounds showed a significant matrix effect, with the exception of TMAO (−34% at 100 ng/mL and −38% at 1000 ng/mL); however, the coefficient of variation (CV) was less than 5% and so can be easily corrected with TMAO-d9. The use of deuterated internal standards made it possible to correct the matrix effect for all the compounds measured. A stability study showed that the analytes were not stable at room temperature (20 °C) for 5 h. In contrast, the analytes were stable during storage in a refrigerator (4 °C) for 24 h or in a freezer (−20 °C) for 3 months. Lastly, an analysis showed that the analytes were not significantly degraded by three freeze/thaw cycles (<15% degradation).

The median concentrations of UT and precursors measured in saliva and serum samples from healthy volunteers are given in Table 2.

Few studies have investigated the concentrations of UTs and amino acids in saliva. Saliva levels of PCS and indoxyl sulfate were measured by Giebultowicz et al. in 70 healthy volunteers [11]. The median (range) PCS level was 22 ng/mL (1.4–362 ng/mL) and the indoxyl sulfate median level was 9.9 ng/mL (2.6–87 ng/mL). These results are consistent with our present findings: PCS = 12 ng/mL (3–69 ng/mL) and indoxyl sulfate = 7 ng/mL (2–56 ng/mL). The mean tyrosine and phenylalanine concentrations measured here (2536 ng/mL and 2047 ng/mL, respectively) were very similar to those reported by Masoudi Rad et al. for 31 healthy controls (2737 ng/mL and 1921 ng/mL, respectively) [12]. Our method was not sensitive enough to detect the physiologic levels of kynurenic acid (<10 ng/mL), kynurenine (<50 ng/mL), or p-cresyl glucuronide (<10 ng/mL). The fact that previously reported kynurenic acid concentrations in healthy controls were very low (mean: 0.6 ng/mL) [13] shows that a dedicated method is necessary. However, the method might be sensitive enough for patients with CKD because they have higher levels in the blood and (probably) in the saliva. To the best of our knowledge, our study is the first to have reported on the saliva concentrations of TMAO, hippuric acid, phenylacetylglutamine, and indole-3-acetic acid.

**Figure 1:**
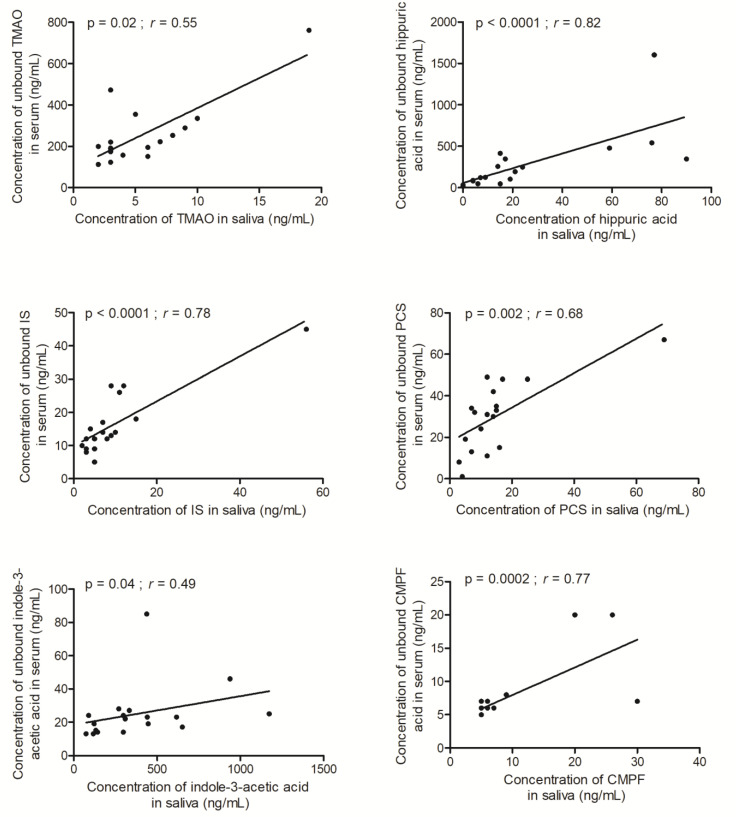
Correlation between saliva and free serum concentrations of trimethylamine-N-oxide (TMAO), hippuric acid, indoxyl sulfate (IS), p-cresyl sulfate (PCS), indole-3-acetic acid, and 3-carboxy-4-methyl-5-propyl-2-furanpropanoic acid (CMPF).

We observed statistically significant correlations between the saliva and free serum and the concentrations for TMAO (r = 0.55), hippuric acid (r = 0.82), indoxyl sulfate (r = 0.78), PCS (r = 0.68), indole-3-acetic acid (r = 0.49), and CMPF (r = 0.77) (Figure 1). A strong correlation between the PCS and indoxyl sulfate was described previously by Korytowska et al. [9]. This kind of relationship has also been observed previously for an exogenous substance (phenytoin) and endogenous substances (cortisol and creatinine) [5,6,7]. Our results suggest that at least TMAO, hippuric acid, indoxyl sulfate, PCS, indole-3-acetic acid, and CMPF have the potential to become routinely assayed in saliva as a proxy for the evaluation of the non-protein-bound fraction in serum.

Significant saliva–serum correlations were not observed for tyrosine (r = −0.43), phenylalanine (r = −0.11), tryptophan (r = −0.30), and phenylacetylglutamine (r = 0.21). For these compounds, the serum concentrations were higher than the saliva concentrations. This difference might be due to degradation by the mouth’s microbiota and/or salivary enzymes or a lack of transmembrane transport from the blood to the saliva via the OAT transporters [12,13,14,15]. Our method must now be tested on samples from CKD, in order to establish whether these very simple assays could be used to monitor UT levels and the related toxicity.

## 3. Conclusions

The present study is the first to have described the saliva concentrations of TMAO, hippuric acid, phenylacetylglutamine, and indole-3-acetic acid. Although we did not study samples from patients with CKD, the saliva concentrations could be compared with the serum free fraction, and a significant correlation was observed for six UTs: TMAO, hippuric acid, indoxyl sulfate, PCS, indole-3-acetic acid, and CMPF. The method did not allow us to determine correlations for three UTs (kynurenine, kynurenic acid, and p-cresyl glucuronide) because the concentrations were below the limit of detection in many samples. These results should prompt further research into the value of saliva samples for monitoring UT levels, particularly in CKD patients.

## 4. Materials and Method

### 4.1. Chemicals and Reagents

Sigma Aldrich (St Quentin Fallavier, France) provided indole-3-acetic acid, indoxyl sulfate, hippuric acid, kynurenic acid, TMAO, phenylalanine, kynurenine, indole-3-acetic acid-d5, tryptophan, tyrosine, sodium hydrogen phosphate, formic acid, and dimethylsulfoxide (DMSO). Phenylacetyl-L-glutamine, phenylacetyl-L-glutamine-d5, PCS, kynurenine-d4, p-cresyl-sulfate-d7, p-cresyl glucuronide, hippuric acid-d5, p-cresyl glucuronide-d7, TMAO-d9, tyrosine-d4, tryptophan-d5, phenylalanine-d5, kynurenic acid-d5, and 3-carboxy-4-methyl-5-propyl-2-furan were provided by Toronto Research Chemicals (North York, Toronto, ON, Canada). VWR Chemicals (Radnor, PA, USA) supplied the sodium dihydrogen phosphate and sodium chloride. Fisher Scientific (Illkirch, France) provided the LC-MS-grade methanol, water, and acetonitrile.

### 4.2. Stock Solution Preparation

Stock solutions were prepared at a concentration of 1 g/L and stored at −20 °C. Working solutions were prepared at four concentrations (100, 10, 1, and 0.1 µg/mL) and stored at −20 °C. The deuterated internal standards were pooled in a methanolic solution and stored at −20 °C. Calibration standards were prepared by spiking 50 μL of distilled water and were treated in the same way as the samples.

### 4.3. Method

#### 4.3.1. Sample Collection

Saliva and blood samples were collected simultaneously in 18 healthy volunteers after the provision of informed consent. The 1 mL saliva sample was collected (in the absence of stimulation) between 9 am and 12 midday by spitting into a plastic tube. Samples were immediately frozen and stored at –80 °C until the day of the analysis.

Blood samples were collected in serum-separating tubes (Vacutainer^TM^, Becton Dickinson and Company, Franklin Lakes, NJ, USA) and immediately centrifuged. The serum samples were stored at −80 °C until the day of analysis.

#### 4.3.2. Sample Preparation

- Saliva

After addition of 25 μL of internal standard solution, 50 μL of saliva were precipitated with 340 μL of methanol. After homogenization and centrifugation for 10 min at 9000× *g* at 4 °C, the supernatant was evaporated, and the dry residue was reconstituted with 80 μL of water and injected into the chromatography system.

- Serum

Serum samples were processed according to a previously published method [10]. Briefly, free UT levels were measured after separation of the unbound fraction with ultra-centrifugal filters (30 kDa, 0.5 mL, Amicon, Merck, Darmstadt, Germany). One hundred and fifty μL of serum were placed in the ultra-centrifugal filter and then centrifuged at 13,300 rpm for 20 min. The residual filtrate was treated in the same way as the saliva residue.

#### 4.3.3. The LC-MS/MS Apparatus

The analysis was performed on an UltiMate™ 3000 LC system (Thermo Fisher Scientific, Les Ulis, France) equipped with a degasser, an auto-sampler, and a binary pump coupled to a tandem mass spectrometer (TSQ Quantiva, Thermo, Les Ulis, France). Nitrogen was used for nebulization and argon was used as the collision gas.

The compounds were eluted on an Accucore^TM^ PFP column (100 × 2.1 mm, 2.6 μm, Thermo Fisher Scientific) maintained at 40 °C. The mobile phase A consisted of water with 0.1% formic acid, and the mobile phase B was acetonitrile. The flow rate was 500 µL/min. The mobile phase B gradient was as follows: 0 min 1%; 1 min 1%; 6.5 min 65%; 6.6 min 90%; 8 min 90%; 8.1 min 1%. UTs were detected using the TSQ Quantiva spectrometer. The ionization mode, the multiple reaction monitoring transitions, the cone voltage, and the collision energies are given in Supplementary Document S2. Data were acquired and processed using Xcalibur software (version 4.2.28.14, Thermo Fisher Scientific).

#### 4.3.4. Method Validation

UTs are endogenous and so calibration standards and quality controls cannot be prepared directly from human serum or saliva. Wakamatsu et al. suggested using water as a blank matrix, in order to quantify endogenous substances with mass spectrometry; this strategy has already been applied by Itoh et al. for UT quantification and by our group in a previous publication [10,16,17]. Here, we applied the approach that had been used previously to quantify UTs in serum. Thus, the calibration and quality control methods were exactly the same for UTs in saliva vs. serum. Only two parameters had to be validated: the matrix effect and sample stability (Supplementary Document S1).

- The matrix effect

UTs are naturally present in biological matrices and so it is not possible to assess the matrix effect by overloading saliva with these compounds. Given that the deuterated internal standards have the same physical–chemical properties as UTs, they were used to evaluate the matrix effect at concentrations of 100 and 1000 ng/mL. At each of the two concentrations, six points were prepared in six saliva samples and another six were prepared in pure water. The matrix effect was defined as the ratio between the mean peak areas in each matrix and the mean peak area obtained in water.

- Stability

The samples’ stability was evaluated by analyzing three samples after 5 h at 20 °C, 24 h at 4 °C, and 24 h at −80 °C and with one, two, or three freeze/thaw cycles. Analytes were considered stable when the end concentration was similar to the initial concentration (CV ≤ 15%).

### 4.4. Statistical Analysis

All statistical analyses were performed with GraphPad Prism software (version 5.0, GraphPad, La Jolla, CA, USA). A non-parametric Spearman test was used to evaluate the correlation between the saliva concentration and the serum concentrations of each UT.

## Figures and Tables

**Table 1:** Limit of detection (LOD), linearity range, and matrix effect for the 10 UTs and the three precursors.

Uremic Toxins (UT)	LOD (ng/mL)	Linearity (ng/mL)	Matrix Effect (CV%) 100 ng/mL *n* = 6	Matrix Effect (CV%) 1000 ng/mL *n* = 6
CMPF	0.3	1–50,000	+21% (10%)	+8% (9%)
Hippuric acid	2	5–50,000	−11% (12%)	−10% (12%)
Indole-3-acetic acid	2	5–50,000	+16% (15%)	+3% (15%)
Indoxyl sulfate	0.3	1–50,000	+4% (11%)	−7% (13%)
Kynurenine	20	50–50,000	−11% (12%)	−6% (14%)
Kynurenic acid	20	10–50,000	−7% (14%)	−4% (13%)
P-cresyl glucuronide	20	10–10,000	−4% (13%)	−5% (13%)
PCS	0.3	1–50,000	+4% (13%)	−2% (11%)
Phenylacetylglutamine	3	10–50,000	+1% (12%)	0% (15%)
Phenylalanine	20	50–50,000	−3% (12%)	−9% (7%)
TMAO	3	1–50,000	−34% (3%)	−38% (5%)
Tryptophan	20	50–50,000	+6% (13%)	−4% (13%)
Tyrosine	20	50–50,000	−5% (11%)	−20% (13%)

LOD: limit of detection; CV: coefficient of variation; CMPF: 3-carboxy-4-methyl-5-propyl-2-furanpropanoic acid; PCS: P-cresyl sulfate; TMAO: trimethylamine-N-oxide.

**Table 2:** Median concentrations measured in saliva and in serum (free fractions), the serum/saliva ratio, Spearman’s correlation coefficient, and the *p*-value for analyses in 18 healthy volunteers.

Molecules	Median (Range) UT Concentration in Saliva (ng/mL)	Median (Range) Concentration of Free UT in Serum (ng/mL)	Serum/Saliva Ratio	Spearman Correlation Coefficient (r)	*p*-Value
TMAO	4 (2–19)	197 (112–761)	51 (24–143)	0.55	0.02
Tyrosine	2536 (976–11,404)	7632 (5022–13,530)	3.1 (0.5–12)	−0.43	0.08
Phenylalanine	2047 (1000–16,624)	8159 (5457–13,933)	4.6 (0.5–11.8)	−0.11	0.66
Kynurenine	<50	< 50 (<50–54)	12.7 (2.6–267)	NF^4^	NF^4^
Tryptophan	143 (<50–1863)	1335 (662–2292)	12 (0.5–108)	−0.30	0.22
Hippuric acid	15 (<5–90)	159 (16–1604)	15 (3.0–78.5)	0.82	<0.0001
Phenylacetylglutamine	11 (<10–20)	203 (22–340)	10 (4.8–17)	0.21	0.41
Indoxyl sulfate	7 (2–56)	14 (5–45)	2.4 (0.8–4.6)	0.78	<0.0001
Kynurenic acid	<10	<10	NF^4^	NF^4^	NF^4^
P-cresyl glucuronide	<10 (<10–12)	10 (<10–57)	4.0 (0.1–48)	NF^4^	NF^4^
PCS	12 (3–69)	31 (1–67)	2.4 (0.4–5.3)	0.68	0.002
Indole-3-acetic acid	302 (74–1174)	22 (13–85)	0.08 (0.02–0.28)	0.49	0.04
CMPF	5 (5–30)	6 (5–20)	0.1 (0.2–1.4)	0.77	0.0002

TMAO: trimethylamine-N-oxide. NF: not feasible; PCS: P-cresyl sulfate; CMPF: 3-carboxy-4-methyl-5-propyl-2-furanpropanoic acid.

## Data Availability

The data presented in this study are available on request from the corresponding author.

## Supplementary Materials

The following supporting information can be downloaded at: https://www.mdpi.com/article/10.3390/toxins15020150/s1, Supplementary Document S1: Validation report; Supplementary Document S2: Molecular mass, chemical formula, retention time (RT), ionization mode, and analytical parameters.
