# Supplementary material for: Correlation between Saliva Levels and Serum Levels of Free Uremic Toxins in Healthy Volunteers
Source: Toxins (Basel). 2023 Feb 13;15(2):150. doi: 10.3390/toxins15020150 (PMC9967652; doi:10.3390/toxins15020150)
Supplement: Supplementary file 1 [file toxins-15-00150-s001.zip › Supplementary document S1.pdf]

Validation report

1. Validation Method

1.1. Linearity

Six calibration curves were prepared with eight calibration standards (CS). Quantification was achieved by plotting the peak area ratios of uremic toxins (UT) to their internal standards (IS), and a weighting factor was determined. Back-calculated concentrations of the CS have to be within 85–115% of the nominal concentrations, except for the lower limit of quantification (LLOQ) a deviation of 20% was considered acceptable.

1.2. Lower Limit of Quantification (LLOQ)

The LLOQ was the lowest concentration of each UT achievable with an accuracy between 80 and 120 % and a coefficient of variation of ± 20 % obtained over six measurements at three different days. Depending to the UT, four levels were evaluated: 1, 5, 10 and 50 ng/mL.

1.3. Carry over

A blank sample was analyzed immediately after running three replicates of the highest CS to determine the carryover.

1.4. Accuracy and precision

The accuracy (bias, %) and precision (coefficient of variation, CV %) of the assay were determined for the four QC levels (30, 150, 8000 and 40000 ng/mL) over three different days. Each day, 6 replicates of each QC were processed. The intra-assay bias and CV takes into account the variability of the 6 replicates within each validation day and the inter-assay bias and CV evaluates the variability between the three validation days. An accuracy within the range 85-115% of the nominal values and a precision with a CV of ±15% were required.

2. Validation results

2.1. Linearity

Table S1. Linearity range, mean correlation coefficient and mean slope.

Molecules	Linearity (ng/mL)	R^2 ± SD	Slope ± SD
TMAO[1]	1-50000	0.9993 ± 0.0004	0.0032 ± 0.0035
Tyrosine	50-50000	0.9992 ± 0.0009	0.0111 ± 0.0055
Phenylalanine	50-50000	0.9986 ± 0.0009	0.0023 ± 0.0003
Kynurenine	50-50000	0.9987 ± 0.0020	0.0064 ± 0.0031
Tryptophan	50-50000	0.9992 ± 0.0003	0.0047 ± 0.0005
Hippuric acid	5-50000	0.9990 ± 0.0006	0.0002 ± 0.00003
Phenylacetylglutamine	10-50000	0.9991 ± 0.0007	0.0154 ± 0.0083
Indoxyl sulfate	1-50000	0.9993 ± 0.0006	0.0140 ± 0.0027
Kynurenic acid	10-50000	0.9992 ± 0.0007	0.0206 ± 0.0038
P-cresyl glucuronide	10-10000	0.9991 ± 0.0009	0.0022 ± 0.0003
PCS[2]	1-50000	0.9995 ± 0.0004	0.1648 ± 0.0355
Indole-3-acetic acid	5-50000	0.9991 ± 0.0003	0.0057 ± 0.0010
CMPF[3]	1-50000	0.9993 ± 0.0007	0.0009 ± 0.0007

[1]Trimethylamine-N-oxide; [2]P-cresyl sulfate; [3]3-carboxy-4-methyl-5-propyl-2-furanpropanoic acid

Table S2. Mean concentration of calibrant, standard deviation, coefficient of variation and bias.

		Theoretical concentrations (ng/mL)							
Molecules		**10**	**50**	**100**	**500**	**1000**	**5000**	**10000**	**50000**
TMAO[1]	Mean (n=6)	10.8	49.3	98.4	484	1020	4771	10125	50088
	SD	0.55	3.48	6.85	30.8	60.4	272	435	491
	CV	5%	7%	7%	6%	6%	6%	4%	1%
	Bias	-7%	1%	2%	3%	-2%	5%	-1%	0%
Tyrosine	Mean (n=6)	-	51.1	91.9	532	987	5040	10013	49840
	SD	-	4.91	9.63	40.3	76.5	136	97.1	48.4
	CV	-	10%	10%	8%	8%	3%	1%	0%
	Bias	-	-2%	8%	-6%	1%	-1%	0%	0%
Phenylalanine	Mean (n=6)	-	48	99.2	515	1065	4977	10307	44407
	SD	-	2.32	5.05	36.1	49.4	359.6	690.6	15980
	CV	-	5%	5%	7%	5%	7%	7%	4%
	Bias	-	5	1%	-3%	-6%	0%	-3%	11%
Kynurenine	Mean (n=6)	10.4	48.6	97.3	491	1026	4929	10117	49764
	SD	0.75	4.32	5.30	39.1	76.4	325	176	430
	CV	7%	9%	5%	8%	7%	7%	2%	1%

	Bias	-4%	3%	3%	2%	-3%	1%	-1%	0%
Tryptophan	Mean (n=6)	11.1	50.3	96.4	502	1010	4862	9928	50338
	SD	0.70	3.58	6.41	56.1	38.5	222	540	656
	CV	6%	7%	7%	11%	4%	5%	5%	1%
	Bias	-11%	-1%	4%	0%	-1%	3%	1%	-1%
Hippuric acid	Mean (n=6)	-	53.5	103	476	977	4748	10255	50322
	SD	-	1.81	6.77	34.0	50.2	262	304	662
	CV	-	3%	7%	7%	5%	6%	3%	1%
	Bias	-	-7%	-4%	5%	2%	5%	-3%	-1%
Phenylacetylglutamine	Mean (n=6)	10.8	48.9	95.7	477	1046	4854	10151	50240
	SD	1.21	3.50	4.92	30.2	77.1	236	451	731
	CV	11%	7%	5%	6%	7%	5%	4%	1%
	Bias	-8%	2%	4%	5%	-5%	3%	-2%	0%
Indole-3-acetic acid	Mean (n=6)	-	53.3	96.6	486	1012	4889	10115	50248
	SD	-	2.29	5.49	42.7	35.9	285	573	525
	CV	-	4%	6%	9%	4%	6%	6%	1%
	Bias	-	-7%	3%	3%	-1%	2%	-1%	0%
p cresyl glucuronide	Mean (n=6)	9.99	47.6	101	503	1039	4987	10102	-
	SD	1.09	1.80	8.31	42.3	62.7	224	298	-
	CV	11%	4%	8%	8%	6%	4%	3%	-
	Bias	1%	5%	-1%	-1%	-4%	0%	-1%	-
CMPF[2]	Mean (n=6)	10.4	50.9	95.8	481	1029	4944	10129	50169
	SD	0.56	4.52	5.46	42.8	35.7	250	240	759
	CV	5%	9%	6%	9%	3%	5%	2%	2%
	Bias	-4%	-2%	4%	4%	-3%	1%	-1%	0%
Indoxyl sulfate	Mean (n=6)	10.1	50.2	99.1	491	1032	4966	10299	49866
	SD	1.64	4.54	7.83	38.7	73.9	338	209	422
	CV	16%	9%	8%	8%	7%	7%	2%	1%
	Bias	-1%	0%	1%	2%	-3%	1%	-3%	0%
Kynurenic acid	Mean (n=6)	10.1	48.2	101	495	1043	4914	10095	50308
	SD	0.95	3.91	5.00	41.1	52.1	283	432	666
	CV	9%	8%	5%	8%	5%	6%	4%	1%
	Bias	-1%	4%	0%	1%	-4%	2%	-1%	-1%
PCS[3]	Mean (n=6)	9.91	48.9	97.3	515	1028	4983	10052	49944
	SD	0.78	3.21	6.19	34.2	40.8	302	313	496.
	CV	8%	7%	6%	7%	4%	6%	3%	1%
	Bias	1%	2%	3%	-3%	-3%	0%	-1%	0%

[1]Trimethylamine-N-oxide; [2]3-carboxy-4-methyl-5-propyl-2-furanpropanoic acid; [3]P-cresyl sulfate.

2.2. Lower Limit of Quantification

Table S3. Lower limit of quantification (LLOQ).

		Intra-day (n=6)		Inter-day (n=6)	
Molecules	LLOQ (ng/mL)	CV (%)	Bias (%)	CV (%)	Bias (%)
TMAO[1]	1	12.5	11	7.8	14.9
Tyrosine	50	8.5	-3.4	10.0	-2.2
Phenylalanine	50	4.4	-5.8	5.0	5.4
Kynurenine	50	6.8	0.0	7.0	3.8
Tryptophan	50	5.1	-6.8	6.0	-3.0
Hippuric acid	5	11.3	-0.4	8.6	3.3
Phenylacetylglutamine	10	10.7	-18.0	11.0	-12.0
Indoxyl sulfate	1	16.2	2.7	10.9	-1.0
Kynurenic acid	10	6.3	0.0	9.0	-1.0
P-cresyl glucuronide	10	13.5	1.0	11.0	-2.0
PCS	1	6.4	6.2	14.0	9.9
Indole-3-acetic acid	5	8.5	-7.6	19.6	2.5
CMPF[3]	1	3.5	7.5	7.2	11.2

[1]Trimethylamine-N-oxide; [2]P-cresyl sulfate; [3]3-carboxy-4-methyl-5-propyl-2-furanpropanoic acid

2.3. Carry over

No chromatographic peaks were evident for the analytes and for the internal standards when a blank was injected just after the highest point in the calibration.

2.4. Accuracy and precision

Table S4. Intra-day and inter-day accuracy and precision.

		Intra-day repeatability (n = 6)		Inter-day repeatability (n = 6)	
Molecules	Theoretical concentration (ng/mL)	CV (%)	Bias (%)	CV (%)	Bias (%)
TMAO[1]	30	3.3	16.7	7.9	3.3
	150	2.4	-3.3	7.5	-0.7
	8000	1.6	-8.0	7.8	-0.7
	40000	6.9	5.7	5.2	-5.0
Tyrosine	30	< LOQ			
	150	10.5	12.0	10.8	-2.7
	8000	4.1	-3.1	5.2	-0.4
	40000	6.5	9.4	4.1	-11.1
Phenylalanine	30	< LOQ			
	150	8.6	-3.3	9.1	-2.7
	8000	2.0	-10.2	5.3	-4.0
	40000	5.0	10.4	4.9	1.6
Kynurenine	30	< LOQ			
	150	4.3	3.3	8.8	1.3
	8000	1.9	-12.4	6.6	-3.5
	40000	5.0	-5.1	7.9	-4.9
Tryptophan	30	< LOQ			
	150	7.2	10.0	13.0	-4.7
	8000	4.7	-7.9	9.0	-0.2
	40000	3.8	11.0	7.5	-0.3
Hippuric acid	30	11.8	6.7	7.7	-0.7
	150	5.8	6.7	5.2	2.0
	8000	5.8	4.3	5.1	-2.5
	40000	3.1	10.8	5.7	-3.1
Phenylacetylglutamine	30	12.6	10.0	9.0	6.7
	150	5.9	2.0	5.6	3.3
	8000	5.3	-0.5	10.6	-1.8
	40000	6.2	7.9	9.2	-0.2
Indoxyl sulfate	30	8.8	3.3	3.3	1.3
	150	4.3	6.7	5.9	3.3
	8000	10.1	-1.7	7.0	-5.1
	40000	7.6	6.7	10.9	-2.2
Kynurenic acid	30	4.8	13.3	8.1	3.3
	150	2.5	-3.3	6.6	0.0
	8000	2.0	-9.7	7.9	-1.4
	40000	4.4	8.0	4.1	-2.0
p-cresyl glucuronide	30	12.3	6.7	10.2	3.3
	150	6.5	-4.0	2.4	-2.0
	8000	4.1	0.8	2.8	-2.9
	40000	> limit of linearity			
PCS[2]	30	7.8	10.0	4.4	10.0
	150	4.9	0.0	6.6	-1.3
	8000	7.0	2.4	8.0	-2.2
	40000	8.1	1.9	6.4	-1.8
Indole-3-acetic acid	30	4.4	-3.3	7.2	-1.0
	150	3.5	5.3	8.7	2.7
	8000	3.1	-7.8	9.6	-5.1
	40000	8.3	1.6	7.4	-2.2
CMPF[3]	30	12.8	3.3	8.1	-6.7
	150	4.1	3.3	7.0	0.7
	8000	3.3	-10.0	8.5	-1.5
	40000	4.2	9.6	7.6	-3.3

[1]Trimethylamine-N-oxide; [2]P-cresyl sulfate; [3]3-carboxy-4-methyl-5-propyl-2-furanpropanoic acid
