# Supplementary material for: Correlation between Saliva Levels and Serum Levels of Free Uremic Toxins in Healthy Volunteers
Source: Toxins (Basel). 2023 Feb 13;15(2):150. doi: 10.3390/toxins15020150 (PMC9967652; doi:10.3390/toxins15020150)
Supplement: Supplementary file 1 [file toxins-15-00150-s001.zip › Supplementary document S2.pdf]

Supplementary document S2. Molecular mass, chemical formula, retention time (RT), ionization mode, and analytical parameters.

Compounds	Molecular weight (g/mol)	Chemical formula	Retention time (min)	Ionization Mode (ESI)	Parent ion (*m*/*z*)	Fragment ion (*m*/*z*)	Collision Energy (V)
TMAO	75.1	C_3H_9NO	0.60	+	76.4	58.2<br>59.2	19<br>12
Tyrosine	181.1	$C_9H_{11}NO_3$	0.78	+	182.1	136.1<br>165.0	30<br>20
Phenylalanine	165.1	$C_9H_{11}NO_2$	1.57	+	166.2	120.1<br>103.0	14<br>27
Kynurenine	208.1	$C_{10}H_{12}N_2O_3$	1.87	+	213.1	98.2<br>146.4	15<br>19
Tryptophan	204.1	$C_{11}H_{12}N_2O_2$	4.75	+	205.1	188.1<br>146.0	10<br>17
Hippuric acid	179.1	$C_9H_9NO_3$	4.75	-	178.1	77.1<br>131.8	17<br>21
Phenylacetyl-glutamine	264.1	$C_{13}H_{16}N_2O_4$	4.82	+	270.1	130.1<br>136.1	15<br>15
p-cresyl glucuronide	284.1	$C_{13}H_{16}O_7$	5.08	-	283.1	107.1<br>113.1	31<br>12
Kynurenic acid	189.0	$C_{10}H_7NO_3$	5.63	+	190.1	116.0<br>190.1	31<br>19
Indoxyl sulfate	213.0	$C_8H_7NO_4S$	5.75	-	212.1	80.0<br>81.0	23<br>17
Indole-3-acetic acid	175.1	$C_{10}H_9NO_2$	6.00	+	176	130.1<br>103.1	16<br>31
p-cresyl sulfate	188.0	$C_7H_8O_4S$	6.18	-	187.1	107.1<br>105.1	21<br>34
CMPF	240.1	$C_{12}H_{16}O_5$	7.05	-	239.1	195.1<br>151.1	12<br>16
